# Kounis syndrome: acute myocardial injury triggered by ant bite-induced anaphylaxis

**DOI:** 10.1093/omcr/omaf020

**Published:** 2025-04-28

**Authors:** Omar Altermanini, Anas A Ashour, Waleed K Abdullatef, Abdulrahman Arabi, Mhd Baraa Habib

**Affiliations:** College of Medicine, QU Health, Qatar University, P.O. Box 2713, Doha, Qatar; Internal medicine department, Hamad medical corporation, P.O. Box 3050, Doha, Qatar; Cardiology department, Heart hospital, Hamad medical corporation, P.O. Box 3050, Doha, Qatar; Cardiology department, Heart hospital, Hamad medical corporation, P.O. Box 3050, Doha, Qatar; Cardiology department, Heart hospital, Hamad medical corporation, P.O. Box 3050, Doha, Qatar

**Keywords:** Kounis syndrome, anaphylaxis, acute myocardial injury, ant bite

## Abstract

Anaphylaxis triggered by insect bites is well-documented, but its association with acute myocardial injury (AMI) is rare. We report a case of Kounis syndrome, where an ant bite induced anaphylaxis and myocardial injury. A 47-year-old diabetic male presented with anaphylaxis following an ant bite, exhibiting hypotension, respiratory distress, and wheezing. Electrocardiography revealed transient widespread ST depression and ST elevation in lead aVR, a pattern reflecting global ischemia, which is uncommon in Kounis syndrome. Treatment with intramuscular adrenaline stabilized his condition. Peak troponin T levels were 1306 ng/l. Coronary angiography and cardiac MRI were unremarkable, ruling out significant coronary artery disease. The patient was diagnosed with Kounis syndrome and discharged with an EpiPen and instructions for anaphylaxis management. This case highlights the potential for ant bites to trigger anaphylaxis-associated myocardial injury, emphasizing the importance of prompt diagnosis and management of Kounis syndrome in similar scenarios.

## Introduction

Kounis syndrome (KS), also known as acute myocardial injury (AMI) secondary to allergic response, was initially introduced by Kounis and Zavras in 1991 [1]. Later in 2016, it was redefined to include the simultaneous occurrence of AMI with mast cell and platelet activation in the context of anaphylactic insult, allergy, or hypersensitivity [2]. KS is not known to be rare; rather, it is known to be underreported and underdiagnosed. Therefore, the prevalence of the syndrome is still not well established [2]. A nationwide analysis conducted in the United States on patients hospitalized for allergic symptoms found that KS was reported in 1.1% of the patients [3]. All-cause mortality rate among patients reported to have KS was 7% [3]. The etiology of this syndrome includes any trigger that leads to an allergic reaction. This includes causes such as food, medications (non-steroidal anti-inflammatories, antibiotics), and insect stings [4]. Herein, we present the case of a middle-aged man who developed anaphylaxis following a black ant bite, subsequently leading to AMI.

## Case report

**Figure 1:**
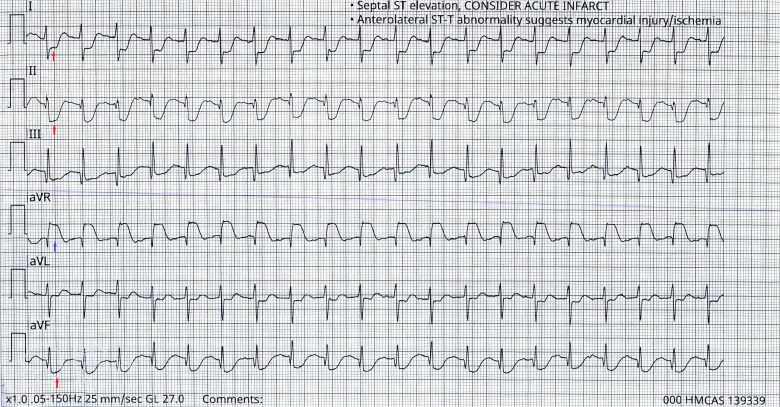
Limb leads electrocardiogram showing diffuse ST depression with ST elevation in aVR. Arrows in Lead I, II, and aVF indicate ST depression and arrow in aVL indicates ST elevation.

**Figure 2:**
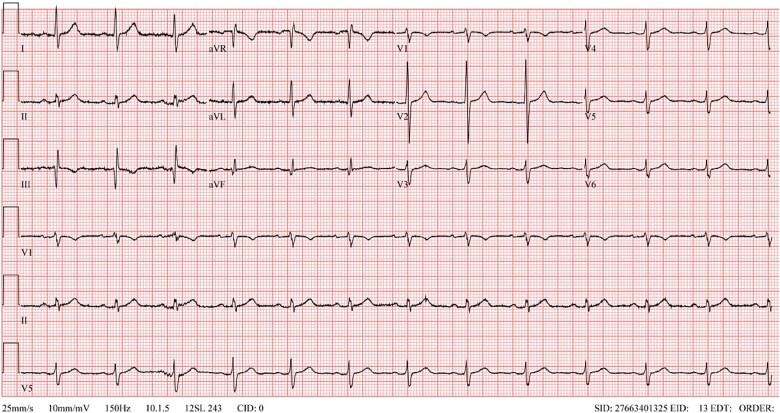
Post-treatment electrocardiogram showing resolution of the changes observed on presentation.

A 47-year-old gentleman presented to the emergency department with dyspnea and rashes following a black ant bite. The symptoms started shortly after the bite and were associated with dizziness. He denied having chest pain or loss of consciousness. No other significant complaints. The patient has previous episodes of anaphylaxis due to ant bites and bee stings requiring adrenaline injections. Other medical history is only remarkable for type-2 diabetes mellitus managed with oral medications. The patient was found hypotensive and received two intramuscular adrenaline dosages of 0.5 mg, spaced five minutes apart after which his condition improved. His vital signs were a temperature of 36.6 C, a blood pressure of 115/75 mmHg, a respiration rate of 20 breaths/minute, and a pulse rate of 80 beats/minute. Physical examination was noncontributory except for minor erythema around the location of the ant bite with no wheezing or weak peripheral pulses. Electrocardiogram (ECG) was done and showed diffuse ST depression with ST elevation in aVR (Fig. 1). Initial blood workup revealed a white blood cell count of 13 500/ul, a troponin-T of 52 ng/l (reference value < 15 ng/l), and a lactic acid level of 9.4 mmol/L (Table 1). The patient was observed for three hours and symptomatically stable. A repeat troponin-T was performed and showed a level of 1306 ng/l. The patient was admitted under cardiology service as a case of non-ST elevation MI. He was started on aspirin, clopidogrel, atorvastatin, and heparin infusion. He underwent a Coronary angiogram which revealed patent coronary arteries with an overall slow blood flow in all arteries consistent with Kounis syndrome. Following that, Cardiac magnetic resonance imaging (MRI) was done to evaluate for possible myocarditis or cardiomyopathy which yielded an unremarkable finding. Throughout the course of hospital admission, the patient remained symptomatically stable with troponin-T levels gradually declining and subsequent resolution of the initial ECG changes (Fig. 2). The final diagnosis was found to be anaphylaxis with non-ST elevation myocardial injury. The patient was discharged with aspirin, atorvastatin, and epinephrine self-injector pen with instructions for anaphylaxis management.

**Table 1:** Initial laboratory investigations of the patient.

Test (unit)	Labs	Normal range
Hemoglobin (gm/dl)	15.5	13–17
White blood cells (× 10^3^/ul)	13.5	4–10
Platelets (× 10^3^/ul)	285	150–410
Creatinine (umol/l)	104	62–106
Sodium (mmol/l)	144	133–146
Potassium (mmol/l)	3.3	3.5–5.3
C-reactive protein (mg/l)	6.4	< 5
Troponin-T HS (ng/l)	1306	3–5
Cholesterol (mmol/l)	4.6	< 5.2
Triglyceride (mmol/l)	1.1	< 1.7
HDL (mmol/l)	1.2	
LDL (mmol/l)	2.9	< 2.59
HbA1C (%)	8.3	< 5.7
Lactic Acid (mmol/l)	9.4	0.5–2.2

## Discussion

Kounis syndrome encompasses a spectrum of acute myocardial syndromes triggered by allergic or hypersensitivity reactions. It is classified into three subtypes based on the underlying mechanism [5]. Type 1 KS occurs in patients without prior coronary artery disease, where an allergic reaction induces coronary artery spasm. Type 2 KS occurs in patients with pre-existing inactive atheromatous plaques, where hypersensitivity reactions lead to plaque erosion or rupture. Type 3 KS is associated with drug-eluting coronary stent thrombosis in the setting of an allergic response [5].

The pathophysiology of Kounis syndrome involves complex interactions between hypersensitivity reactions and the cardiovascular system. A key mechanism is the activation of mast cells by various triggers, leading to the release of vasoactive substances such as histamine [6]. These mediators induce coronary vasospasm, platelet activation, and, in some cases, coronary thrombosis [6]. Clinically, KS patients often present with overlapping features of an allergic reaction and acute coronary syndrome (ACS) [7]. Symptoms may include hypotension, urticaria, rash, dyspnea, and wheezing, alongside anginal chest pain and fatigue mimicking ACS [7]. Diagnosing KS is challenging due to its dual cardiovascular and anaphylactic components, which are often overlooked or underdiagnosed. The initial workup should include ECG and cardiac markers. Additionally, cardiac angiography is often required when initial tests are suspicious of ACS. If angiography was not definitive of a vascular insult, cardiac MRI could be useful to exclude other causes.

In our case, the patient demonstrated features of Type 1 KS, as there was no prior history of coronary artery disease. Interestingly, the patient’s ECG revealed transient widespread ST depression with ST elevation in lead aVR, which was also associated with significant troponin-T trend. This pattern is uncommon in KS, as most published cases describe inferior wall changes [8–11]. The observed ECG findings likely reflect global ischemia and diffuse coronary vasospasm, which caused left main coronary artery ischemia—a mechanism supported by histamine and other inflammatory mediators in KS [6].

A review article of 235 KS cases identified antibiotics (32.3%) and non-steroidal anti-inflammatory drugs (24.3%) as the most common triggers [12]. KS triggers vary significantly, for example, certain drugs can induce type-1 hypersensitivity reactions that involve mast cell degranulation and histamine release, resulting in coronary vasospasm [13]. Several case reports highlight different insect bites as triggers for KS. One case described a fire ant bite causing Type 1 KS via mast cell degranulation induced by venom in a patient with no coronary heart disease [14]. Another case involved a bee sting in a patient with pre-existing atherosclerosis, resulting in a combination of Type 1 and Type 2 KS due to plaque rupture and vasospasm [11]. Additionally, a case series described two cases triggered by a caterpillar and a paper wasp, where both patients experienced rash and developed ACS three to six days later [15]. This presentation contrasts with our case, in which ACS occurred only a few hours after the black ant bite. In our case, the black ant bite triggered severe mast cell activation, leading to coronary vasospasm within minutes to hours, unlike the delayed reactions noted in the prior cases. This rapid progression could be explained by the direct introduction of allergens or vasoactive chemicals in the venom, which may exacerbate vasospasm.

Currently, there are no standardized guidelines for Kounis syndrome management. For Type 1 KS, the priority is controlling the allergic response, as this can resolve the coronary vasospasm without further treatment of the cardiac event. However, the other types of KS require treatment of the cardiac event that aligns with standard ACS protocols, focusing on addressing plaque rupture and thrombosis. Further studies are necessary to establish comprehensive treatment protocols for this syndrome.

## Conclusion

Ant bites can induce anaphylaxis, which may lead to myocardial injury with ST segment changes. When anaphylaxis is suspected or diagnosed, it is crucial for physicians to assess and monitor for potential cardiac complications, given the serious nature of the condition and the absence of established treatment protocols. Management should focus on treating the anaphylactic reaction while observing the hemodynamic status and managing the coronary event.

## Data Availability

Data sharing is not applicable to this article as no datasets were generated or analyzed during the current study.
